# Editorial: Application of meta-omics in biotechnology, environmental monitoring, and health

**DOI:** 10.3389/fgene.2023.1229830

**Published:** 2023-06-12

**Authors:** Grace Nkechinyere Ijoma

**Affiliations:** Department of Environmental Sciences, College of Agricultural and Environmental Sciences, University of South Africa, Roodepoort, South Africa

**Keywords:** meta-omics, biomarkers, biotechnology, transcriptomics, public health

The advancement of meta-omics and its integration into a variety of fields of biotechnology, environmental monitoring, and health over the last decade has been astounding and very promising. Our quest to develop personalized medicine and even improve human quality of life owes a great deal to meta-omics. Thus, the purpose of this Research Topic was to attract prospective and unique research trends and breakthroughs in meta-omics technologies relevant to biotechnological processes, environmental monitoring, and health. The six accepted articles delivered on this goal with intriguing discoveries that will shape our approach to applying meta-omics to the area of biomedical research in the coming years.

Interestingly, cell line research work done by Dave et al. revealed a novel approach, by applying single cell line model systems while capturing all represented populations and subpopulations, exposing their diverse and unique functional characteristics, which may have been lost with previous approaches. They provide an automated cell identification platform to be used before single-cell culturing that lowered cell stress and improved rare cell identification. These researchers’ pipeline can be utilized for patient samples to reduce processing time while retaining data quality and accuracy towards understanding the patient’s disease. This contribution to high-throughput single-cell multi-data assays makes it easier to quantify therapeutic responses, classify differentiation heterogeneity in generated models, and validate single-cell sequencing conclusions, which helps understand disease pathogenesis and tumor metastasis.

In their review paper, Li et al. noted that spatial transcriptomics’ localization-indexed gene expression data could help understand cancer’s complex heterogeneity. These researchers criticized single-cell sequencing for the limited information, it tends to provide. Remarkably, the previous research group of Dave et al. appeared to have been working on this challenge highlighted by Li et al. Both articles will be interesting to readers as they provide unintentional supporting contexts and contribute to breast cancer research in a great way. Li et al. also underline the importance of transcriptomics in tumor heterogeneity research and personalized therapy. Through comprehensive research, the authors demonstrate that intra-tumor heterogeneity hinders cancer diagnosis and personalized treatment. These scientists also note that novel technologies may be expensive, but they believe spatial omics, particularly spatial transcriptomics, will provide the most insights for screening tumor treatment targets and improving clinical diagnosis accuracy.

Boileau et al. created the Single-cell Nanopore Spatial Transcriptomics (SCNAST), a new software suite that demonstrates the capability to analyze spatial gene expression from second- and third-generation sequencing platforms, to create a full-length near-single-cell transcriptional landscape of the tissue microenvironment. Their product development applied Visium Spatial to assign barcodes to long-read single-cell sequencing data for spatial capture technology. They successfully created a *de novo* transcriptome (long-read data) of a mouse heart after a myocardial infarction using four short axis heart sections. The successful assignment of 19,794 transcript isoforms revealed the spatial diversity of isoforms, a fascinating information that can help create new heart failure and cardiac problems treatments.

The study by Zhang et al. highlights the utility of RNA-seq platforms for investigating molecular pathways in Dermatofibrosarcoma protuberans (DFSP), a rare cutaneous sarcoma. These researchers used differential expression and enrichment analysis to tackle fourteen DFSP samples from Chinese patients. They were successful in identifying DFSP fusion genes, biomarkers, and microenvironment traits. Their study reveals the efficacy of RNA-seq as a viable technique for getting new insights into disease, leading to accurate diagnosis and specificity in therapeutic targets for certain diseases like DFSP.

Differential co-expression network analysis continues to be useful in meta-omics research, as demonstrated by Kasavi, who used this strategy to identify novel candidate biomarker signatures in three datasets of serous ovarian adenocarcinoma. The researcher discovered 439 common differentially expressed genes (DEGs) and created differential co-expression networks utilizing common DEGs in two situations, in this case healthy ovarian surface epithelia and serous ovarian cancer epithelia. This type of study, in particular, allowed for the likely identification of novel candidate predictive biomarkers for ovarian cancer. The latter is suggested to be potentially useful in ovarian cancer diagnosis and treatment advances.

Finally, Zhou et al. investigated the link between metabolic reprogramming and malignant tumors. Their review focused on the non-essential amino acid serine and its role in cell proliferation. Their extensive literature survey highlighted the potential use of serine as a biomarker in tumor detection and pathology. They showed the importance serine metabolism plays in the network of glycolysis, folate cycle, and one-carbon metabolic pathways. These researchers, also suggest that future studies should target the fate of serine in these varied pathways for both normal functioning cells and diseased cells as this will be critical in the development of precision and personalized cancer therapy.

